# Population genetics of mouse lemur vomeronasal receptors: current versus past selection and demographic inference

**DOI:** 10.1186/s12862-017-0874-6

**Published:** 2017-01-21

**Authors:** Philipp Hohenbrink, Nicholas I. Mundy, Ute Radespiel

**Affiliations:** 1 Institute of Zoology, University of Veterinary Medicine Hannover, Buenteweg 17, 30559 Hannover, Germany; 2 Department of Zoology, University of Cambridge, Downing St, Cambridge, CB2 3EJ UK

**Keywords:** VNO, Evolution, Madagascar, *Microcebus murinus*, *Microcebus ravelobensis*, V1R, V2R, Genetic diversity, Demography, Selection

## Abstract

**Background:**

A major effort is underway to use population genetic approaches to identify loci involved in adaptation. One issue that has so far received limited attention is whether loci that show a phylogenetic signal of positive selection in the past also show evidence of ongoing positive selection at the population level. We address this issue using vomeronasal receptors (VRs), a diverse gene family in mammals involved in intraspecific communication and predator detection. In mouse lemurs, we previously demonstrated that both subfamilies of VRs (V1Rs and V2Rs) show a strong signal of directional selection in interspecific analyses. We predicted that ongoing sexual selection and/or co-evolution with predators may lead to current directional or balancing selection on VRs. Here, we re-sequence 17 VRs and perform a suite of selection and demographic analyses in sympatric populations of two species of mouse lemurs (*Microcebus murinus* and *M. ravelobensis*) in northwestern Madagascar.

**Results:**

*M. ravelobensis* had consistently higher genetic diversity at VRs than *M. murinus.* In general, we find little evidence for positive selection, with most loci evolving under purifying selection and one locus even showing evidence of functional loss in *M. ravelobensis*. However, a few loci in *M. ravelobensis* show potential evidence of positive selection. Using mismatch distributions and expansion models, we infer a more recent colonisation of the habitat by *M. murinus* than by *M. ravelobensis,* which most likely speciated in this region earlier on.

**Conclusions:**

These findings suggest that the analysis of VR variation is useful in inferring demographic and phylogeographic history of mouse lemurs. In conclusion, this study reveals a substantial heterogeneity over time in selection on VR loci, suggesting that VR evolution is episodic.

**Electronic supplementary material:**

The online version of this article (doi:10.1186/s12862-017-0874-6) contains supplementary material, which is available to authorized users.

## Background

Adaptation leaves its mark in the genome. These genomic signatures of adaptation can be investigated using phylogenetic and population genetic approaches, which address the different evolutionary timescales of interspecific and intraspecific variation. A rapidly growing body of literature is documenting the loci involved in adaptation at one of these levels [1]. However, there are few studies which directly evaluate the relationship between the two, or separate studies examining the same loci at different timescales (for example, genes involved in brain development in primates [2, 3]). Hence it is an open question whether positive selection that occurs at loci among species is generally reflected in positive selection at the same loci within species.

Different selection regimes make different predictions for the relationship between past and ongoing selection. In cases where there is an ongoing co-evolutionary dynamic, such as in host-parasite or Red Queen systems, selection on key loci may be more or less continuous and a signal of selection is expected both in the past and present. Under balancing selection, for example, multiple alleles may be under on-going frequency-dependent selection across speciation events, and similar signals of selection can therefore be expected to act from the past to the present. Another possibility is that directional selection during a certain evolutionary period leads to the fixation of novel and advantageous variants. After this first and potentially episodic period of adaptation, purifying selection may then follow to stabilize and maintain the acquired adaptations. Under these conditions, there will be discordance between inferences of past and ongoing selection. Many other patterns are also possible: for example, changes in the effective population size (N_e_) will affect fixation of mildly deleterious alleles by drift and selection on compensatory alleles, so changes in N_e_ alone may lead to different signatures of selection in different timeframes [4].

In mammals, G-protein coupled chemoreceptors form one of the gene families that are most consistently found to be under positive selection in phylogenetic analyses [5]. One of the most diverse classes of chemoreceptors in terrestrial mammals is vomeronasal receptors (VRs) that function in intraspecific communication and predator detection. There is evidence for positive selection on both types of VR (V1R and V2R) in mammals [6, 7]. This is consistent with the possibility of arms races between the scent of predators and the VRs of their prey, or the action of sexual selection on VR evolution for intraspecific communication.

Mouse lemurs (*Microcebus*) are nocturnal strepsirrhine primates with an elaborated olfactory behavioural repertoire [8–10]. Olfactory stimuli play an important role in their intraspecific communication as well as in predator detection [11–14]. They have the largest repertoire of VR genes (~200 *V1R*s and 2 *V2R*s) of any primate. We previously showed a high level of positive selection acting on multiple families of *V1R* genes, as well as *V2R* genes, during mouse lemur evolution [15, 16]. We hypothesised that this positive selection may relate to predator detection and/or intraspecific communication. If this is the case then one would also expect current selection on mouse lemur VR loci. To the best of our knowledge, population genetics of VRs has not previously been investigated in the wild.

Functional loci have been relatively neglected in studies of demography, which favour neutral markers (e.g., [17–21]). The phylogeography of many taxa has been influenced by Pleistocene glaciation cycles, and this has been demonstrated in temperate regions as well as in the tropics (e.g., [22, 23]). For Madagascar with its highly complex ecogeography of central-eastern highlands, a dry western and a humid eastern zone and various seasonal forest types, various biogeographic hypotheses have been proposed that take into account Pleistocene climatic changes, topographic barriers such as mountains and large rivers [24, 25]. It has been suggested that various recent endemic radiations in lemurs and other vertebrates are causally linked to these climatic fluctuations that led to cyclic vegetation changes within and across isolated centres of endemism [26].

Mouse lemurs (*Microcebus* spp.) are a highly diverse genus with currently 24 described species [27] that are mostly confined to rather local or regional geographic ranges that are separated from each other by large rivers. Allopatric speciation has therefore been proposed as predominant mechanism of diversification within this genus [28, 29]. However, one species, the grey mouse lemur (*Microcebus murinus*), occurs from southern Madagascar up to northwestern Madagascar and can be found in partial sympatry with at least five other local or regional mouse lemur species, *M. griseorufus, M. berthae* *, M. myoxinus, M. ravelobensis* and *M. bongolavensis* (reviewed in [30]). Two recent molecular studies suggest that this large geographic range is the result of a rather recent expansion from southern to northwestern Madagascar at some time point in the late Pleistocene [21, 31]. Schneider et al. [21] inferred this expansion based on modelling and simulating the diversity and gene genealogy of mitochondrial sequence data obtained from several populations in northwestern Madagascar which also included samples from the same study site as chosen for this study. The haplotype diversity sampled in that study could be best explained by a succession of two spatial expansions that could be dated to the late Pleistocene (younger than 350,000 years old). It was hypothesised that an ancestral population of *M. murinus* colonized this region (=inter-river-system, IRS) before the last glacial maximum (LGM) and then contracted into riverine refugia during the dry period that coincided with the LGM and presumably with the preceding glacial periods in Madagascar [26]. From there it may subsequently have expanded again in parallel with forest expansion [21]. In the second study, Blair et al. [31] inferred the rather recent expansion of *M. murinus* by modelling the split between *M. murinus* and its sister species, *M. griseorufus*, in southwestern Madagascar. Coalescent methods were employed on 55–124 sequences for four molecular markers (alpha enolase intron, alpha fibrinogen intron, von Willebrand factor intron, cytochrome B concatenated with cytochrome c oxidase subunit II), respectively, stemming from localities in southern to western Madagascar. Results were concordant with allopatric speciation from a narrowly distributed common ancestor that lived in southwest Madagascar. Only *M. murinus* underwent a subsequent range expansion to the north and experienced severe population dynamics during the Pleistocene [31]. Given these scenarios, the demographic history of *M. murinus* populations in northwestern Madagascar, the region which it last colonized, should be substantially different from those of their sympatric congeners that most likely evolved in those areas over longer timescales [28, 32].

Here we assay sequence variation in 15 *V1R* and two *V2R* loci in populations of the two sympatric mouse lemur species (*M. murinus* and *M. ravelobensis*) from northwest Madagascar. We perform various tests of neutrality and investigate the correlation between past and present selective pressures. In addition, we perform demographic analyses to assess the suitability of VR loci for making demographic inferences.

## Methods

### Data collection

We extracted DNA from 20 grey mouse lemurs, *M. murinus* (10 male, 10 female), and 20 golden-brown mouse lemurs, *M. ravelobensis* (10 male, 10 female, see Additional file 1 for details), using a phenol-chloroform protocol and a REPLI-g WGA kit (Qiagen). Small ear biopsies were collected between May and October 2008 in the Ankarafantsika National Park in northwestern Madagascar by S. Thorén (authorisation no. 062/08/MEEFT/ SG/DGEF/DSA/SSE). All animals were live-trapped in the 30.6 ha study site JBA (46°48′E, 16°19′S; for details about the trapping procedure see [33]), and we selected individuals from different trapping locations to minimise the risk of sampling several individuals from the same sleeping group who are most likely related [34, 35]. All sampled animals of the same species are defined to belong to the same population.

We designed locus-specific primer pairs for 15 *V1R* and both *V2R* loci using the online software Primer3Plus [36]. All loci (see Table 1 for details) are expressed in the VNO of the grey mouse lemur [37]. Previous work on these loci in mouse lemurs provided no evidence for paralogues or recent duplicates [15, 16]. We used twelve loci from seven of the nine monophyletic *V1R* clusters and three unclustered loci [16]. Clusters I and VIII were not analysed because locus-specific primers could not be successfully designed for these. The PCR amplicons of intronless *V1R* loci covered the whole locus as all primers bind outside of the coding sequence. *VN2R1* consists of six exons and because of long intron sequences between the exons we designed exon-specific external primers. In contrast to our first description of *V2R*s [15], *VN2R2* consists of only five exons. Previously, we had used cDNA of extracted RNA without introns and sequenced a fragment spanning exon 3 to 5 according to closely related *V2R*s of family D in mice. A short fragment (22 bp) between these exons was assigned to exon 4. However, the present study which uses external primers reveals that the 22 bp of exon 4 rather belongs to exon 3 which is longer in mouse lemurs than in mice. The analogous exon 4 of mice is missing in mouse lemurs, which is in concordance with genomic data of the two strepsirrhines *Daubentonia madagascariensis* and *Otolemur garnettii* where no “exon 4” was detected previously (see [15]), but a similar 22 bp insertion at the end of exon 3 was in fact present. In the present study we could not design external primers for exon 2 of *VN2R2* because of missing genomic data. Due to internal primers we are missing 108 bp (=13.2%) of the sequence of exon 2 and therefore results on *VN2R2* are based on 95.7% of the whole coding sequence.

**Table 1:** *V1R* and *V2R* loci analysed with corresponding gene cluster according to Hohenbrink et al. [16] and total length

Locus	Cluster	Length
*VN1R Mmur001*	IV	909 bp
*VN1R Mmur011*	VI*	930 bp
*VN1R Mmur031*	V*	909 bp
VN1R Mmur033*	uncl	942 bp
*VN1R Mmur040*	II*	948 bp
*VN1R Mmur041*	V*	906 bp
*VN1R Mmur043*	VI*	1005 bp
VN1R Mmur048*	VI*	957 bp
*VN1R Mmur049*	VII*	879 bp
VN1R Mmur060*	V*	906 bp
*VN1R Mmur065*	uncl	1008 bp
VN1R Mmur066*	uncl	897 bp
*VN1R Mmur067*	III	921 bp
VN1R Mmur074*	IX*	918 bp
*VN1R Mmur075*	IV	909 bp
*VN2R1*	V2R	2739 bp
VN2R2*	V2R	2310 of 2418 bp^a^

^a^ = locus 3 bp longer in *M. ravelobensis,* *: Loci and clusters under significant positive selection [15, 16], *uncl* unclustered

We used MyTaq DNA polymerase for amplification (Bioline; 25 μl total volume containing 5.0 μl MyTaq Reaction Buffer, 1 μl of each primer [10 μM stock concentration], 0.1 μl Taq DNA polymerase [5 U/μl] and 1 μl of DNA) with the following PCR conditions: 94 °C for 2 min, 40 times (94 °C for 30 s, 60 °C for 45 s, 72 °C for 90 s), 72 °C for 5 min. PCR products were sequenced on both strands using BigDye Terminator 3.1 (Applied Biosystems) under standard conditions and run on an Applied Biosystems 3500 capillary sequencing machine. Consensus sequences were built with SeqMan 5.05 (DNASTAR Inc., Madison, WI, USA). Sequences were aligned and analysed using MEGA 5 [38]. Before data analyses we concatenated the exons of the *V2R*s.

### Data analyses

We used DnaSP 5.10 [39] to unphase the two alleles of each diploid sequence and to identify the different haplotypes. Nucleotide diversity and haplotype diversity (expected heterozygosity, or gene diversity, [40]) were calculated with DnaSP 5.10 to estimate the genetic variation within the population. DnaSP 5.10 was used to estimate the number of polymorphic synonymous (*p*_S_) and nonsynonymous substitutions (*p*_N_) per site [41, 42] and *p*_N_/*p*_S_ ratios were calculated. McDonald-Kreitman tests (= MKT, [43]) were conducted online [44] to calculate the ratio of fixed differences to polymorphic differences for synonymous and nonsynonymous SNPs. Here, all haplotypes of *M. murinus* and the most closely related haplotype sequence of *M. ravelobensis* were entered to test for positive selection in *M. murinus*, and vice versa to test in *M. ravelobensis*.

Arlequin 3.5 [45] was used to calculate Tajima’s *D* and Fu’s *Fs* with 1000 simulated samples. Population contractions or balancing selection can result in significant positive values of Tajima’s *D*, whereas negative values indicate population growth [46, 47]. Fu’s *Fs* show negative values if the data contains an excess of rare haplotypes also indicating population growth and/or positive selection [48]. In combination, positive *D* and positive *Fs* values indicate an excess of intermediate-frequency alleles after population subdivision or balancing selection, whereas negative *D* and negative *Fs* values reflect relative excess of rare variants and reveal population growth [49]. We used False Discovery Rate (FDR) to correct for multiple testing across both species for each analysis, setting FDR to 0.05 [50].

We analysed the distribution of nonsynonymous SNPs along the *V1R* protein [transmembrane, extra- or intracellular region; for details see 16]. Observed vs. expected χ^2^-tests were used to compare the observed distribution of nonsynonymous SNPs in the *V1R* protein with the expected distribution using Statistica 6.1 (StatSoft, Inc., Tulsa, OK). A previous study on *V1R*s in strepsirrhines reported that the ligand binding site of the *V1R* protein is potentially formed by about half of the 4 and 5th transmembrane region and the in-between 2nd extracellular loop (= 3rd extracellular region) [51]. This estimation was based on VR sequence data of cluster I only, but assuming structural similarities between clusters, we also tested if nonsynonymous substitutions were concentrated on the binding site proposed by Yoder et al. [51]. All statistical comparisons of dependent data between the two species were conducted with the Wilcoxon Matched Pairs test in Statistica. Here, the sample size was large enough to ignore *p*-value corrections that would have been necessary for smaller sample sizes [52]. No such analyses were performed for V2Rs since there is relatively little structural information available for them.

Arlequin 3.5 was also used to test two expansion models (demographic and spatial expansion) on the mismatch distributions of the haplotypes within this single population of each species that span the same spatial scale. A mismatch distribution is a distribution of the number of nucleotide mismatches between all pairs of nucleotide sequences of one locus within a given sample. For this study each individual (homozygous or heterozygous) always entered two sequences into the data pool. Mismatch distributions can be directly compared between loci or species in this study, because of the same sampling regime (see above), same spatial spread of the samples, and the same sample size of 40 nucleotide sequences per locus and species. The shape of a mismatch distribution has been shown to be influenced by demographic events like past expansions or population bottlenecks [53]. Mismatch distributions are bell-shape (= unimodal) in populations having increased in the past as a consequence of one demographic [53] or spatial expansion [54, 55] or L-shaped in case of a very recent size reduction [53]. A previous modelling approach [21] revealed that two successive expansions can generate a tri-modal mismatch distribution and the position of these modes corresponds to the time of the expansion. In contrast, populations at demographic equilibrium show a more ragged distribution [53] (= multimodal). Demographic expansions usually result from past genetic bottlenecks, whereas spatial expansions usually follow a colonisation event by relatively few founder individuals. We tested the expansion models available in Arlequin 3.5 to evaluate the evidence for a preceding colonisation event [54, 56]. The models also calculate *τ*-(*Tau*-) values that reflect the time of the expansion (in mutation units, *τ* = 2*Tμ*), although exact time points are difficult to infer as reliable mutation rates are often not known. However, higher values of *τ* indicate that the expansion happened further in the past.

## Results

### Comparison of genetic diversity

For *V1R*, almost all comparisons showed substantially higher genetic diversity in *M. ravelobensis* than *M. murinus* (Table 2). Across all loci, *M. ravelobensis* possessed significantly more polymorphic sites than *M. murinus* (Wilcoxon-Test, *n* = 15, *Z* = 3.04, *p* < 0.01), more haplotypes (Wilcoxon-Test, *n* = 15, *Z* = 3.15, *p* = 0.001), higher nucleotide diversity (Wilcoxon-Test, *n* = 15, *Z* = 2.78, *p* = 0.005), higher haplotype diversity (Wilcoxon-Test, *n* = 15, *Z* = 2.44, *p* = 0.015) and higher numbers of protein alleles (Wilcoxon-Test, *n =* 15, *Z* = 2.76, *p* = 0.006, Table 2). All alleles at all loci had open reading frames of the expected length except locus *Mmur040* in *M. ravelobensis*, where an allele with an internal stop codon, indicating pseudogenization, occurred at a frequency of 6/40 = 0.15.

**Table 2:** Measures of genetic diversity for each locus and species

	Number of haplotypes		No. diff. AA sequences		Nucleotide diversity		Haplotype diversity		No. of poly-morphic sites	
Locus	Mmur	Mrav	Mmur	Mrav	Mmur	Mrav	Mmur	Mrav	Mmur	Mrav
*001*	7	10	3	8	.00073	.00493	.528	.787	4	13
*011*	3	4	3	3	.00194	.00061	.600	.442	4	3
*031*	5	9	5	5	.00141	.00194	.686	.708	6	9
*033*	3	9	2	9	.00089	.00445	.472	.877	3	15
*040*	6	10	5	10	.00074	.00420	.592	.622	5	21
*041*	4	9	2	4	.00141	.00202	.727	.745	4	10
*043*	4	5	3	3	.00048	.00070	.377	.503	4	4
*048*	11	9	6	3	.00170	.00072	.785	.474	10	9
*049*	2	9	2	8	.00033	.00180	.296	.819	1	9
*060*	3	9	2	6	.00016	.00265	.145	.732	2	14
*065*	1	2	1	1	.00000	.00014	.000	.142	0	1
*066*	4	17	3	14	.00139	.00560	.558	.954	5	28
*067*	2	14	1	9	.00015	.00417	.142	.859	1	15
*074*	6	12	6	10	.00093	.00475	.487	.777	8	25
*075*	7	9	1	6	.00098	.00506	.668	.709	7	21
Ø V1R	4.5	9.1	3.0	6.6	.00088	.00292	.471	.677	4.3	13.1
*VN2R1*	25	28	24	25	.00332	.00268	.973	.976	29	32
*VN2R2*	14	27	8	20	.00053	.00220	.838	.967	16	28
Ø V2R	19.5	27.5	16	22.5	.00193	0.0024	.906	.972	22.5	30

*diff. AA sequences:* number of different amino acid sequences, *Mmur: M. murinus*, *Mrav: M. ravelobensis*, Ø *V1R/V2R:* mean

For both species, there were more haplotypes at *V2R* loci than *V1R* loci (Table 2). However, the number of polymorphic sites in *V2R*s was increased more dramatically in *M. murinus* than in *M. ravelobensis*. The number of unique amino acid sequences was considerably lower than the number of haplotypes in *VN2R2*, but this was not the case in *VN2R1* indicating that here most haplotypes differed by at least one nonsynonymous substitution. Overall, the genetic diversity in *V1R*s and *VN2R2* differs between the species, whereas it was equally high in *VN2R1* of both species.

### Tests of neutrality

Several loci in both species had significantly negative neutrality tests using uncorrected p-values (Table 3). However, using FDR, only three loci remained significant, all for Fu’s *Fs* in *M. ravelobensis* (*Mmur048*, *VN2R1*and *VN2R2*). No significantly positive values were found and test values did not differ significantly between the species (Tajima’s *D*: *n* = 15, *Z* = 0, *p* = 1.000; Fu’s *F*_*s*_: *n* = 15, *Z* = 0.68, *p* = 0.500).

**Table 3:** Results of the neutrality tests, and *p*_N_/*p*_S_ ratios for each locus and species

	Tajima’s *D*				Fu’s *Fs*				*p*_N_/*p*_S_	
Locus	Mmur	p	Mrav	p	Mmur	p	Mrav	p	Mmur	Mrav
*001*	−0.70	0.287	1.46	0.939	−3.83	0.005	0.78	0.649	0.19	0.18
*011*	2.18	0.978	−0.42	0.398	3.96	0.941	−0.70	0.286	0.32	0.87
*031*	−0.24	0.469	−0.48	0.341	0.36	0.605	−2.05	0.147	0.91	0.11
*033*	0.42	0.700	0.60	0.763	1.42	0.806	1.21	0.743	0.20	1.13
*040*	−1.02	0.170	0.76	0.828	−2.38	0.055	2.13	0.815	0.29	2.53
*041*	0.86	0.804	−0.66	0.294	1.37	0.794	−1.91	0.160	0.11	0.35
*043*	−1.25	0.107	−0.74	0.272	−1.25	0.167	−1.49	0.134	2.43	0.05
*048*	−1.13	0.137	−1.98	0.007	−4.48	0.011	6.69	*0.000	0.39	0.08
*049*	0.37	0.807	−0.70	0.283	0.84	0.497	−2.44	0.103	—	0.53
*060*	−1.30	0.072	−0.86	0.222	−2.03	0.012	−0.89	0.362	0.15	0.14
*065*	0.00	1.000	−0.56	0.252	0.00	1.000	−0.22	0.209	—	0.00
*066*	0.16	0.608	−0.50	0.358	1.29	0.755	−2.34	0.218	0.31	1.62
*067*	−0.56	0.252	0.28	0.673	−0.22	0.218	−2.63	0.152	0.00	0.59
*074*	−1.56	0.024	−0.87	0.226	−1.77	0.102	−0.63	0.443	0.17	0.45
*075*	−1.27	0.094	−0.23	0.471	−2.69	0.051	1.61	0.776	0.00	0.24
*VN2R1*	1.15	0.914	−0.08	0.526	−6.78	0.022	−13.58	*0.000	0.19	0.49
*VN2R2*	−1.39	0.072	−0.78	0.249	−6.33	0.004	−17.25	*0.000	0.28	0.36

*: *p* < 0.05 with FDR of q = 0.05; *Mmur: M. murinus*, *Mrav: M. ravelobensis*, *—: p*_S_ was zero

### Tests for selection and distribution of mutations across VR proteins

The *p*_S_ of *V1R*s was significantly higher than *p*_N_ in both species (*M. murinus*: *n* = 15, *Z* = 2.92, *p* = 0.004; *M. ravelobensis*: *n* = 15, *Z* = 2.39, *p* = 0.017, Table 3). The *p*_N_/*p*_S_ ratios of *V1R*s did not differ significantly between species (Table 3; *n* = 13, *Z* = 1.01, *p* = 0.311). Using uncorrected p-values, McDonald-Kreitman Tests (MKT) were only significant for *VN1R Mmur066* in *M. ravelobensis* (*p* = 0.026, Table 4), but this result was not robust to multiple testing correction using FDR. There was no significant correlation between the *d*_N_/*d*_S_ of loci estimated across *Microcebus* species [16] and the *p*_N_/*p*_S_ ratios determined intraspecifically in the current study (*M. murinus*: *n* = 5 loci, *r*_*s*_ = 0.5, n.s.; *M. ravelobensis*: *n* = 5 loci, *r*_*s*_ = 0.0, n.s.).

**Table 4:** Results of MK tests and mismatch distributions for each locus and species

	MK		τ_demo_				τ_spat_			
Locus	Mmur p	Mrav p	Mmur	p	Mrav	p	Mmur	p	Mrav	p
*001*	0.538	0.379	0.75	0.47	8.63	0.09	0.75	0.35	6.28	0.33
*011*	0.599	0.998	5.02	0.11	0.58	0.84	3.85	0.13	0.56	0.68
*031*	0.272	0.597	1.88	0.69	3.82	0.88	1.65	0.81	0.18	0.79
*033*	0.051	0.821	0.00	*0.00	6.47	0.20	1.77	0.33	4.86	0.38
*040*	0.140	0.393	0.88	0.10	11.69	0.01	0.87	0.08	9.11	0.43
*041*	0.711	0.873	1.50	0.82	2.61	0.02	1.51	0.82	2.49	0.12
*043*	0.090	0.397	0.48	0.70	0.74	0.84	0.48	0.47	0.73	0.69
*048*	0.135	0.779	1.77	0.86	0.98	0.91	1.01	0.84	0.83	0.86
*049*	0.531	0.731	2.98	0.18	1.48	0.19	0.38	0.08	1.49	0.10
*060*	0.995	0.869	3.00	0.36	3.95	0.27	0.10	0.33	3.06	0.85
*065*	-	0.334	0.00	-	0.21	0.39	0.00	-	0.16	0.35
*066*	0.704	0.026	2.82	0.11	8.28	0.57	2.24	0.10	5.69	0.18
*067*	0.493	0.107	0.21	0.52	7.27	0.61	0.16	0.33	3.80	0.39
*074*	0.449	0.142	3.73	0.49	6.63	0.05	0.05	0.41	5.37	0.22
*075*	0.263	0.399	1.00	0.10	8.00	0.02	1.01	0.04	6.80	0.53
*VN2R1*	0.935	0.225	0.48	0.27	0.74	0.69	0.48	0.16	0.73	0.39
*VN2R2*	0.887	0.344	2.54	0.36	4.46	0.74	2.46	0.56	3.83	0.81

*: *p* < 0.05 with FDR of q = 0.05; *Mmur: M. murinus*, *Mrav: M. ravelobensis*, *τ*_*demo*_: τ-values for demographic expansion model, *τ*_*spat*_: τ-values for spatial expansion model, —: incalculable because of absence of variation

The distribution of nonsynonymous SNPs in the domains of the *V1R* protein did not differ significantly from the expected distribution in any species when looking at the data of all *V1R* loci combined (*M. murinus*: *χ* ^2^ = 3.2, df = 2, *p* = 0.200; *M. ravelobensis*: *χ* ^2^ = 1.0, df = *2*, *p* = 0.606). Similarly, no single locus showed a significant deviation from the expected distributions (for example, *VN1R Mmur066*, the only locus with significant MKT: *χ* ^2^ = 2.6, df = 2, *p* = 0.268; all other loci also with *p* > 0.05). A proposed odorant binding site [51] comprised about 20% of the *V1R* protein. There was no evidence for concentration of NS mutations in this region: across all loci, 21.9% of *M. murinus* NS substitutions and 19.7% of *M. ravelobensis* NS substitutions occurred here, and no single locus showed an excess of NS substitutions in this region.

### Mismatch distributions

**Fig. 1:**
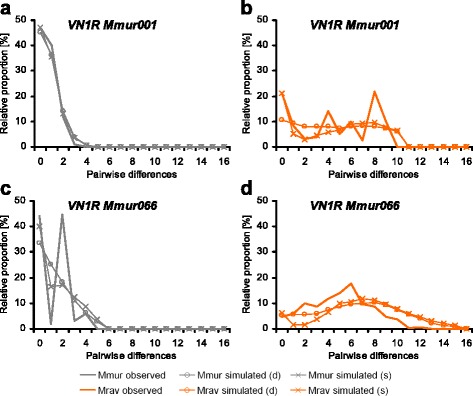
Observed and simulated mismatch distributions of *VN1R Mmur001* and *066* in *M. murinus* (Mmur, left side, *grey*) and *M. ravelobensis* (Mrav, right side, *orange*); the two loci were selected to show the three observed types of distributions: half-bell shaped (**a**), unimodal (**c** + **d**) and ragged (**b**); mismatch distributions of the remaining loci are shown in Additional file 2; simulated (d) = simulated under demographic expansion model (line with *circles*), simulated (s) = simulated under spatial expansion model (line with crosses)

The mismatch distributions showed huge variation between loci and species (Fig. 1 and Additional file 2). In *M. murinus* most *V1R* loci (*n* = 11) had half-bell shaped distributions close to zero pairwise differences or the peak was at zero (Fig. 1a, Table 5, Additional file 2). Furthermore, *M. murinus* had four loci with unimodal distributions (*VN1R Mmur011*, *033*, *066* and *074*) but no locus with multimodal or ragged distributions (Fig. 1c). In contrast, three loci in *M. ravelobensis* showed ragged mismatch distributions (*VN1R Mmur001*, *040*, and *067*, see Fig. 1b) and six had mostly broad unimodal distributions (*VN1R Mmur033*, *041*, *060*, *066*, *074* and *075,* see Fig. 1d). The remaining six *V1R*s showed half-bell shaped distributions similar to *M. murinus*. Notably, unimodal distributions in *M. murinus* were still close to zero pairwise differences with a high peak, whereas they were generally broader and flat in *M. ravelobensis* (compare Fig. 1c + d).

**Table 5:** Pairwise occurrence of the three types of observed mismatch distributions for the *V1R* loci in both species

		*M. murinus*			
		HB	UM	RG	Σ
**M. ravelobensis**	**HB**	5	1	0	6
	**UM**	3	3	0	6
	**RG**	3	0	0	3
	**Σ**	11	4	0	15

*HB:* half-bell (L-) shape distribution, *UM:* unimodal distribution, *RG:* ragged distribution, *Σ:* Sum

The patterns of occurrence of half-bell shaped, unimodal and ragged distributions in the two species are summarized in Table 5. About half of the loci (8 of 15) had similar types of distributions in both species, but six of the remaining mismatch distributions showed higher variation in *M. ravelobensis* than in *M. murinus*. In *V2R*s (Additional file 2), the mismatch distributions in *M. murinus* showed a ragged distribution for *VN2R1* and a unimodal distribution for *VN2R2*. The distributions in *M. ravelobensis* were unimodal for both *V2R*s.

Using uncorrected p-values, tests of demographic expansion were significant in one locus of *M. murinus* (VN1R *Mmur033*, *p* = 0.00) and three *V1R* loci of *M. ravelobensis* (*VN1R Mmur040*: *p* = 0.01; *VN1R Mmur041*: *p* = 0.02; *Mmur075*: *p* = 0.02), and tests of spatial expansion were significant in one locus of *M. murinus* (*Mmur075*: *p* = 0.04, Table 4). However, only one of these results remained significant after FDR correction – demographic expansion for *Mmur033* in *M. murinus*. The τ-values were significantly larger in *M. ravelobensis* compared to *M. murinus* using both models (Table 4; demographic expansion: *n* = 15, *Z* = 2.38, *p* = 0.017; spatial expansion: *n* = 15, *Z* = 2.44, *p* = 0.015) indicating an older starting point of the putative expansion in *M. ravelobensis* compared to *M. murinus*.

## Discussion

### Selection in the recent evolutionary history of vomeronasal receptor genes

It was previously shown that the majority of *V1R* gene clusters in mouse lemurs evolved under strong positive selection and repeated gene duplication led to the evolution of a large *V1R* repertoire [16]. Positive selection still acted on *V1R*s during the diversification of mouse lemurs as indicated by analyses of single *V1R* loci across different mouse lemur species [16] or of a *V1R* subfamily across different lemur species [51]. Positive selection, which was probably involved in generating this high *V1R* diversity in evolutionary timescales, could in principle be ongoing in present-day populations. In contrast to this expectation, the *VR*s we studied seem to be mostly evolving under purifying selection. Several results support the presence of purifying selection at the population level: 1) McDonald-Kreitman tests were mostly non-significant (with one possible exception, see below). 2) Neutrality tests were non-significant in most cases (see below for further discussion). 3) The *p*_N_/*p*_S_ ratio was less than one in most loci of both species. 4) Nonsynonymous substitutions were randomly distributed within the *V1R* protein indicating neutral evolution rather than positive selection. This contrasts with our previous phylogenetic study where replacement substitutions were significantly concentrated at particular parts of the protein, consistent with odorant binding sites [16]. For one locus, *Mmur040* in *M. ravelobensis*, there is even evidence for ongoing loss of function, with an allele encoding a pseudogene segregating at appreciable frequency.

Only few loci showed patterns consistent with current positive selection. *VN1R Mmur066* in *M. ravelobensis* was the only example of a significant MK test with an excess of non-synonymous SNPs segregating in the population, although this was not robust to FDR correction. However, the high haplotype diversity and *p*_N_/*p*_S_ ratio greater than one suggest that the possibility of balancing selection at this locus warrants further investigation. Three loci in *M. ravelobensis* showed significant evidence for departure from neutrality, with a negative Fu’s *Fs* that was robust to FDR correction. The cause of this non-neutrality however is unclear – it may be due to directional selection or population expansion, which was not rejected for these loci from the mismatch distribution tests.

In a previous study we argued that diversification and positive selection on VR loci may be involved in reproductive isolation and speciation of mouse lemurs [16]. If indeed functionally linked to reproduction, it would also be possible that the loci that are potentially under positive selection in the present study could be involved in olfactory mate choice or pheromonal communication in the context of finding a suitable mate (reviewed in [57]). Selection may thus act upon the VRs in the VNO and partly the main olfactory epithelium (MOE) [37]. Although some information is available on the function of certain *V1R* clusters in mice (e.g. [58, 59]), knowledge of the biological function of certain *V1R*s or *V2R*s are lacking completely for primates [16, 51]. In view of the rich and functional *V1R* repertoires of nocturnal strepsirrhines, future studies are urgently needed that shed light on their biological functions. Fruitful future experimental approaches may include behavioural assays involving individuals with polymorphisms at individual loci, and tests of the effect of odorants on the activity of VRs expressed in vitro. By contrast, the use of VNO tissue slices for immunohistochemistry or of anesthetised individuals for electrophysiological recordings, which has been a useful method in rodents [48], is not a viable option for these endangered species.

The higher haplotype diversity for *V2R* genes than *V1R* loci is interesting. The repertoire of *V1R* genes is much more diverse in mouse lemurs than their repertoire of *V2R* genes, with ~200 *V1R* loci [60] and only 2 *V2R* loci currently known in mouse lemurs [15]. It is possible that the paucity of *V2R* genes in the VNO is partly compensated by a high allelic diversity which also translates into a relatively high number of amino acid sequences and could therefore lead to a further increase of the olfactory sensory resolution in the VNO.

The presence of only weak evidence for ongoing selection at VR loci in these two mouse lemur species is in strong contrast to the prevalence of positive selection at the same loci in a comparative study [12]. This is one of the first studies to explicitly compare patterns of positive selection at these two levels, although there have been a few studies in humans [2, 3]. This obvious discordance between the results of the two studies suggests that fixation of beneficial mutations at VRs during mouse lemur evolution may have been highly episodic, i.e. occurring over short periods of time. However, one issue for further consideration is whether the power to detect positive selection is the same in the two approaches. Future studies would be needed to address this issue, for example using simulations.

### Demographic history of two sympatric mouse lemur species

Under the assumption of a divergent phylogeographic history of both mouse lemur species in northwestern Madagascar, we predicted to find differences in the genetic diversity of the VRs between the two species. This prediction was confirmed by several datasets. The species with the longer phylogeographic history in the region, *M. ravelobensis*, possessed significantly more polymorphic sites, a higher number of haplotypes, a higher haplotype and nucleotide diversity, as well as a higher number of different amino acid sequences in its *V1R* loci than its congener with the supposedly shorter phylogeographic history in the region, *M. murinus*. Despite some degree of variability between loci, the results from these functional loci therefore support the conclusion of previous studies about the relatively recent expansion of *M. murinus* into northwestern Madagascar [21, 31] and suggest a distinct founder effect in this species. A similar interspecific difference was visible in the diversity of the two *V2R* loci, although these were generally more diverse in *M. murinus* than the *V1R*s.

Based on the scenario that *M. murinus* colonized northwestern Madagascar only sometime in the late Pleistocene [21, 31], we predicted to find signals of a stronger and/or more recent bottleneck in *M. murinus* than in *M. ravelobensis* which most likely evolved somewhere in this region earlier on [28] and may have maintained a larger ancient effective population size than the expanding founder population of *M. murinus*. As already noted above, although the majority of loci showed negative values in the summary statistics (Tajima’s *D*, Fu’s *Fs*) of both species, which would indicate population expansion under neutrality and/or positive selection, only very few loci deviated significantly from mutation-drift equilibrium. On the other hand, only *M. ravelobensis* showed significantly negative values of Fu’s *Fs* in both *V2R* loci. However, the overall similarity in the patterns of these summary statistics does not support the hypotheses of a largely different demographic history of both species. There may be several reasons for these findings: first, it is possible that these results mostly reflect the most recent demographic history of both species that may have been rather similar in the late Pleistocene forests of northwestern Madagascar. The extent of the forest surface most likely contracted in all western lowland areas of Madagascar towards the last glacial maximum (LGM) and expanded again only afterwards [24–26]. During the LGM, all species, independent on whether they had a long or short phylogeographic history in these lowland forests, probably underwent population contractions and expanded again afterwards together with the forests [21, 26]. In addition, both tested mouse lemur species have most likely been equally affected by the anthropogenic habitat loss that started in large parts of Madagascar after the arrival of man within the last few thousand years and continues until today [61–63]. Second, it is possible that similar selection regimes in the two species acting across multiple loci could affect the allelic diversity of VRs. However, this is unlikely, since there was little evidence for widespread directional selection across VR loci (see above).

In addition to the summary statistics discussed above, mismatch distributions were used to compare the distribution of haplotype diversity within both species. Three types of mismatch distributions were identified in both mouse lemur species, which differed in relative frequency: 1) Half-bell shaped or L-shaped distributions showing a lack of variation which indicates purifying selection or recent bottlenecks [53]; 2) Unimodal distributions that are seen after one demographic or spatial expansion [53]; 3) Ragged distributions that are typical for populations at demographic equilibrium when colonisation events are very old and diversification is not constrained. Although the two species shared the half-bell shape distribution in five loci, *M. ravelobensis* showed the more diverse types of distributions (*n* = 9) more often than *M. murinus* (*n* = 4). In accordance with the results on genetic diversity presented above, these findings are in agreement with the hypothesis of a larger ancient effective population size in *M. ravelobensis* that was able to maintain a larger degree of genetic variability across time than its congener *M. murinus*. The mismatch distributions of both species did not differ significantly from the simulated distribution after one demographic or spatial expansion in most loci (exception: one locus in *M. murinus*). However, the τ-values were significantly higher in *M. ravelobensis* indicating that the putative expansion of *M. ravelobensis* is older than that of *M. murinus*. A more precise estimation of the time since expansion is not possible, since reasonable mutation rates for *VR* loci are not available and evolutionary rates were shown to vary across the entire gene family [16]. Therefore, these species differences cannot be easily reconciled with the present knowledge on certain historic vegetation changes.

## Conclusions

The current VR diversity of *M. murinus* and *M. ravelobensis* in northwestern Madagascar appears to be shaped by various processes such as divergent scenarios of population expansions, purifying selection, loss of function and a potential contribution from positive selection. Whereas strong positive selection, found in the whole *VR* repertoire (both *V1R*s and *V2R*s) and within individual gene clusters occurred in the past [16], ongoing selection may have shifted towards purifying selection in the majority of *V1R* loci to maintain the adaptive function of individual receptors, e.g. in the context of olfactory reproductive isolation between species as well as sex or kairomone recognition. This study only analysed a small subset of the large VR repertoire of mouse lemurs but gives important insights into the recent evolution of VRs and suggests a previously unknown shift in selection pressures acting on these functional genes that are probably of highest relevance for nocturnal solitary foragers that rely heavily on olfactory communication. Functional VR loci may not be best-suited for demographic modelling considering the difficulty of differentiating between signals of purifying selection and recent population bottlenecks. However, the simultaneous analysis of synonymous and nonsynonymous substitutions can help to disentangle these different processes. Future studies on the functional diversity and molecular evolution of olfactory receptor genes will certainly add substantially to our understanding of adaptive radiations, local adaptation and reproductive strategies in various mammalian clades whose life styles, social systems and reproductive strategies rely on pheromonal communication.

## Additional files

Additional file 1: IDs of the sampled individuals; the table contains the IDs of all males and females that were used for sequencing 17 VR loci in the two mouse lemur species *Microcebus murinus* (*n* = 20) and *M. ravelobensis* (*n* = 20) in the study site JBA. (DOCX 14 kb)
Additional file 2: Observed and simulated mismatch distributions of *M. murinus* and *M. ravelobensis* under the demographic and the spatial expansion model; Graphic representation of the 15 mismatch distributions per species that were not included as examples in the main manuscript. The two species-specific distributions for each locus are displayed side by side (Mmur, left side, grey; Mrav, right side, orange). simulated (d) = simulated after demographic expansion model (line with circles), simulated (s) = simulated after spatial expansion model (line with crosses). (PDF 90 kb)

## References

[1] Vitti JJ, Grossman SR, Sabeti PC (2013). Detecting natural selection in genomic data. Annu Rev Genet.

[2] Evans PD, Gilbert SL, Mekel-Bobrov N, Vallender EJ, Anderson JR, Vaez-Azizi LM, Tishkoff SA, Hudson RR, Lahn BT (2005). Microcephalin, a gene regulating brain size, continues to evolve adaptively in humans. Science.

[3] Montgomery SH, Capellini I, Venditti C, Barton RA, Mundy NI (2011). Adaptive evolution of four microcephaly genes and the evolution of brain size in anthropoid primates. Mol Biol Evol.

[4] Mustonen V, Lassig M (2009). From fitness landscapes to seascapes: non-equilibrium dynamics of selection and adaptation. Trends Genet.

[5] Kosiol C, Vinar T, da Fonseca RR, Hubisz MJ, Bustamante CD, Nielsen R, Siepel A (2008). Patterns of positive selection in six Mammalian genomes. Plos Genet.

[6] Mundy NI, Cook S (2003). Positive selection during the diversification of class I vomeronasal receptor-like (V1RL) genes, putative pheromone receptor genes, in human and primate evolution. Mol Biol Evol.

[7] Shi P, Bielawski JP, Yang H, Zhang YP (2005). Adaptive diversification of vomeronasal receptor 1 genes in rodents. J Mol Evol.

[8] Glatston AR, Seth PK (1983). Olfactory communication in the lesser mouse lemur (*Microcebus murinus*). Perspectives in primate biology.

[9] Perret M, Alterman L, Doyle GA, Izard MK (1995). Chemocommunication in the reproductive function of mouse lemurs. Creatures of the dark the nocturnal prosimians.

[10] Schilling A, Doyle GA, Martin RD (1979). Olfactoriy communication in prosimians. The study of prosimian behavior.

[11] Buesching CD, Heistermann M, Hodges JK, Zimmermann E (1998). Multimodal oestrus advertisement in a small nocturnal prosimian, *Microcebus murinus*. Folia Primatol.

[12] Kappel P, Hohenbrink S, Radespiel U (2011). Experimental evidence for olfactory predator recognition in wild mouse lemurs. Am J Primatol.

[13] Perret M, Schilling A (1995). Sexual responses to urinary chemosignals depend on photoperiod in a male primate. Physiol Behav.

[14] Sündermann D, Scheumann M, Zimmermann E (2008). Olfactory predator recognition in predator-naïve gray mouse lemurs (*Microcebus murinus*). J Comp Psychol.

[15] Hohenbrink P, Mundy NI, Zimmermann E, Radespiel U (2013). First evidence for functional vomeronasal 2 receptor genes in primates. Biol Lett.

[16] Hohenbrink P, Radespiel U, Mundy NI (2012). Pervasive and ongoing positive selection in the Vomeronasal-1 Receptor (V1R) repertoire of mouse lemurs. Mol Biol Evol.

[17] Excoffier L (2002). Human demographic history: refining the recent African origin model. Curr Opin Genet Dev.

[18] Kaessmann H, Heissig F, von Haeseler A, Paabo S (1999). DNA sequence variation in a non-coding region of low recombination on the human X chromosome. Nat Genet.

[19] Kawamoto Y, Takemoto H, Higuchi S, Sakamaki T, Hart JA, Hart TB, Tokuyama N, Reinartz GE, Guislain P, Dupain J (2013). Genetic structure of wild bonobo populations: diversity of mitochondrial DNA and geographical distribution. Plos One.

[20] Quach H, Wilson D, Laval G, Patin E, Manry J, Guibert J, Barreiro LB, Nerrienet E, Verschoor E, Gessain A (2013). Different selective pressures shape the evolution of Toll-like receptors in human and African great ape populations. Hum Mol Genet.

[21] Schneider N, Chikhi L, Currat M, Radespiel U (2010). Signals of recent spatial expansions in the grey mouse lemur (*Microcebus murinus*). BMC Evol Biol.

[22] Burney DA, Goodman SM, Benstead JP (2003). Madagascar’s prehistoric ecosystem. The natural history of Madagascar.

[23] Hewitt GM (1999). Post-glacial re-colonization of European biota. Biol J Linn Soc.

[24] Vences M, Wollenberg KC, Vieites DR, Lees DC (2009). Madagascar as a model region of species diversification. Trends Ecol Evol.

[25] Wilmé L, Goodman SM, Ganzhorn JU (2006). Biogeographic evolution of Madagascar’s microendemic biota. Science.

[26] Mercier JL, Wilmé L (2013). The Eco-Geo-Clim model: explaining Madagascar’s endemism. Madagascar Conserv Dev.

[27] Hotaling S, Foley ME, Lawrence NM, Bocanegra J, Blanco MB, Rasoloarison R, Kappeler PM, Barrett MA, Yoder AD, Weisrock DW (2016). Species discovery and validation in a cryptic radiation of endangered primates: coalescent-based species delimitation in Madagascar’s mouse lemurs. Mol Ecol.

[28] Olivieri G, Zimmermann E, Randrianambinina B, Rasoloharijaona S, Rakotondravony D, Guschanski K, Radespiel U (2007). The ever-increasing diversity in mouse lemurs: three new species in north and northwestern Madagascar. Mol Phylogenet Evol.

[29] Weisrock DW, Rasoloarison RM, Fiorentino I, Ralison JM, Goodman SM, Kappeler PM, Yoder AD (2010). Delimiting species without nuclear monophyly in Madagascar’s mouse lemurs. Plos One.

[30] Radespiel U, Lehman SM, Radespiel U, Zimmermann E (2016). Can behavioral ecology help to understand the divergent geographic range sizes of mouse lemurs. The dwarf and mouse lemurs of Madagascar: biology, behavior and conservation biogeography of the cheirogaleidae.

[31] Blair C, Heckman KL, Russell AL, Yoder AD (2014). Multilocus coalescent analyses reveal the demographic history and speciation patterns of mouse lemur sister species. BMC Evol Biol.

[32] Thiele D, Razafimahatratra E, Hapke A (2013). Discrepant partitioning of genetic diversity in mouse lemurs and dwarf lemurs - Biological reality or taxonomic bias?. Mol Phylogenet Evol.

[33] Thorén S, Quietzsch F, Radespiel U (2010). Leaf nest use and construction in the golden-brown mouse lemur (*Microcebus ravelobensis*) in the Ankarafantsika National Park. Am J Primatol.

[34] Radespiel U, Jurić M, Zimmerman E (2009). Sociogenetic structures, dispersal and the risk of inbreeding in a small nocturnal lemur, the golden-brown mouse lemur (*Microcebus ravelobensis*). Behaviour.

[35] Radespiel U, Sarikaya Z, Zimmermann E, Bruford MW (2001). Sociogenetic structure in a free-living nocturnal primate population: sex-specific differences in the grey mouse lemur (*Microcebus murinus*). Behav Ecol Sociobiol.

[36] Untergasser A, Nijveen H, Rao X, Bisseling T, Geurts R, Leunissen JAM (2007). Primer3Plus, an enhanced web interface to Primer3. Nucleic Acids Res.

[37] Hohenbrink P, Dempewolf S, Zimmermann E, Mundy NI, Radespiel U (2014). Functional promiscuity in a mammalian chemosensory system: extensive expression of vomeronasal receptors in the main olfactory epithelium of mouse lemurs. Front Neuroanat.

[38] Tamura K, Peterson D, Peterson N, Stecher G, Nei M, Kumar S (2011). MEGA5: molecular evolutionary genetics analysis using maximum likelihood, evolutionary distance, and maximum parsimony methods. Mol Biol Evol.

[39] Librado P, Rozas J (2009). DnaSP v5: a software for comprehensive analysis of DNA polymorphism data. Bioinformatics.

[40] Nei M (1987). Molecular evolutionary genetics.

[41] Jukes T, Cantor C, Munro HN (1969). Evolution of protein molecules. Mammalian protein metabolism III.

[42] Nei M, Gojobori T (1986). Simple methods for estimating the numbers of synonymous and nonsynonymous nucleotide substitutions. Mol Biol Evol.

[43] McDonald JH, Kreitman M (1991). Adaptive protein evolution at the Adh locus in drosophila. Nature.

[44] Egea R, Casillas S, Barbadilla A (2008). Standard and generalized McDonald-Kreitman test: a website to detect selection by comparing different classes of DNA sites. Nucleic Acids Res.

[45] Excoffier L, Lischer HEL (2010). Arlequin suite ver 3.5: a new series of programs to perform population genetics analyses under Linux and Windows. Mol Ecol Resour.

[46] Tajima F (1989). The effect of change in population-size on DNA polymorphism. Genetics.

[47] Tajima F (1989). Statistical-method for testing the neutral mutation hypothesis by DNA polymorphism. Genetics.

[48] Fu YX (1997). Statistical tests of neutrality of mutations against population growth, hitchhiking and background selection. Genetics.

[49] Bamshad MJ, Mummidi S, Gonzalez E, Ahuja SS, Dunn DM, Watkins WS, Wooding S, Stone AC, Jorde LB, Weiss RB (2002). A strong signature of balancing selection in the 5 ′ cis-regulatory region of CCR5. Proc Natl Acad Sci U S A.

[50] Benjamini Y, Hochberg Y (1995). Controlling the false discovery rate: a practical and powerful approach to multiple testing. J R Stat Soc Series B Stat Methodol.

[51] Yoder AD, Chan LM, dos Reis M, Larsen PA, Campbell CR, Rasoloarison R, Barrett M, Roos C, Kappeler P, Bielawski J (2014). Molecular evolutionary characterization of a V1R subfamily unique to strepsirrhine primates. Genome Biol Evol.

[52] Mundry R, Fischer J (1998). Use of statistical programs for nonparametric tests of small samples often leads to incorrect P values: examples from Animal Behaviour. Anim Behav.

[53] Rogers AR, Harpending H (1992). Population growth makes waves in the distribution of pairwise genetic differences. Mol Biol Evol.

[54] Excoffier L (2004). Patterns of DNA sequence diversity and genetic structure after a range expansion: lessons from the infinite-island model. Mol Ecol.

[55] Ray N, Currat M, Excoffier L (2003). Intra-deme molecular diversity in spatially expanding populations. Mol Biol Evol.

[56] Schneider S, Excoffier L (1999). Estimation of past demographic parameters from the distribution of pairwise differences when the mutation rates very among sites: Application to human mitochondrial DNA. Genetics.

[57] Drea CM (2015). D’scent of man: a comparative survey of primate chemosignaling in relation to sex. Horm Behav.

[58] Isogai Y, Si S, Pont-Lezica L, Tan T, Kapoor V, Murthy VN, Dulac C (2011). Molecular organization of vomeronasal chemoreception. Nature.

[59] Nodari F, Hsu F-F, Fu X, Holekamp TF, Kao L-F, Turk J, Holy TE (2008). Sulfated steroids as natural ligands of mouse pheromone-sensing neurons. J Neurosci.

[60] Young JM, Massa HF, Hsu L, Trask BJ (2010). Extreme variability among mammalian V1R gene families. Genome Res.

[61] Burns SJ, Godfrey LR, Faina P, McGee D, Hardt B, Ranivoharimanana L, Randrianasy J (2016). Rapid human-induced landscape transformation in Madagascar at the end of the first millennium of the Common Era. Quat Sci Rev.

[62] Harper GJ, Steininger MK, Tucker CJ, Juhn D, Hawkins F (2007). Fifty years of deforestation and forest fragmentation in Madagascar. Environ Conserv.

[63] Zinner D, Wygoda C, Razafimanantsoa L, Rasoloarison R, Andrianandrasana HT, Ganzhorn JU, Torkler F. Analysis of deforestation patterns in the central Menabe, Madagascar, between 1973 and 2010. Reg Environ Change. 2013;14:157–66.

