# Supplementary material for: Population genetics of mouse lemur vomeronasal receptors: current versus past selection and demographic inference
Source: BMC Evol Biol. 2017 Jan 21;17:28. doi: 10.1186/s12862-017-0874-6 (PMC5251345; doi:10.1186/s12862-017-0874-6)
Supplement: Additional file 1: — IDs of the sampled individuals; the table contains the IDs of all males and females that were used for sequencing 17 VR loci in the two mouse lemur species Microcebus murinus (n = 20) and M. ravelobensis (n = 20) in the study site JBA. (DOCX 14 kb) [file 12862_2017_874_MOESM1_ESM.docx]

Additional file 1: IDs of the sampled individuals

| *M. murinus* | | *M. ravelobensis* | |
| --- | --- | --- | --- |
| Sex | ID | Sex | ID |
| F | 02-05 | M | 30-06 |
| M | 32-06 | M | 12-07 |
| F | 05-07 | M | 30-07 |
| F | 20-07 | M | 31-07 |
| M | 23-07 | F | 51-07 |
| M | 57-07 | F | 68-07 |
| F | 58-07 | F | 74-07 |
| F | 60-07 | M | 02-08 |
| M | 75-07 | M | 10-08 |
| F | 01-08 | F | 14-08 |
| M | 08-08 | M | 41-08 |
| F | 13-08 | F | 42-08 |
| M | 17-08 | M | 44-08 |
| M | 24-08 | F | 49-08 |
| F | 28-08 | F | 51-08 |
| M | 34-08 | M | 52-08 |
| F | 35-08 | F | 68-08 |
| M | 57-08 | M | 69-08 |
| M | 64-08 | F | 70-08 |
| F | 67-08 | F | 72-08 |

F = female; M = male
