# Supplementary material for: Population genetics of mouse lemur vomeronasal receptors: current versus past selection and demographic inference
Source: BMC Evol Biol. 2017 Jan 21;17:28. doi: 10.1186/s12862-017-0874-6 (PMC5251345; doi:10.1186/s12862-017-0874-6)
Supplement: Additional file 2: — Observed and simulated mismatch distributions of M. murinus and M. ravelobensis under the demographic and the spatial expansion model; Graphic representation of the 15 mismatch distributions per species that were not included as examples in the main manuscript. The two species-specific distributions for each locus are displayed side by side (Mmur, left side, grey; Mrav, right side, orange). simulated (d) = simulated after demographic expansion model (line with circles), simulated (s) = simulated after spatial expansion model (line with crosses). (PDF 90 kb) [file 12862_2017_874_MOESM2_ESM.pdf]

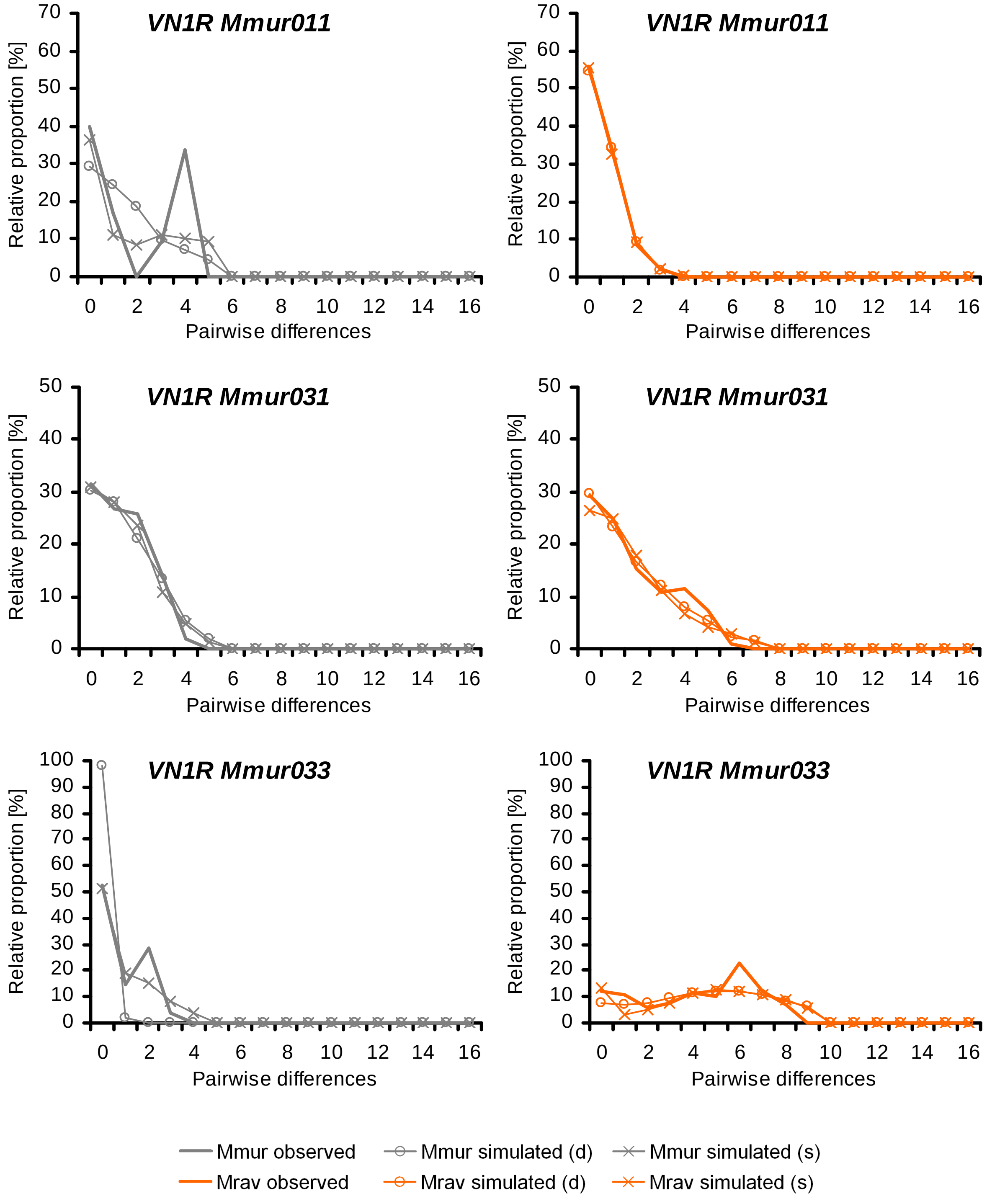

Additional file 2: Observed and simulated mismatch distributions of *M. murinus* (Mmur, left side, grey) and *M. ravelobensis* (Mrav, right side, orange); simulated (d) = simulated after demographic expansion model (line with circles), simulated (s) = simulated after spatial expansion model (line with crosses); (*continues on next pages*)

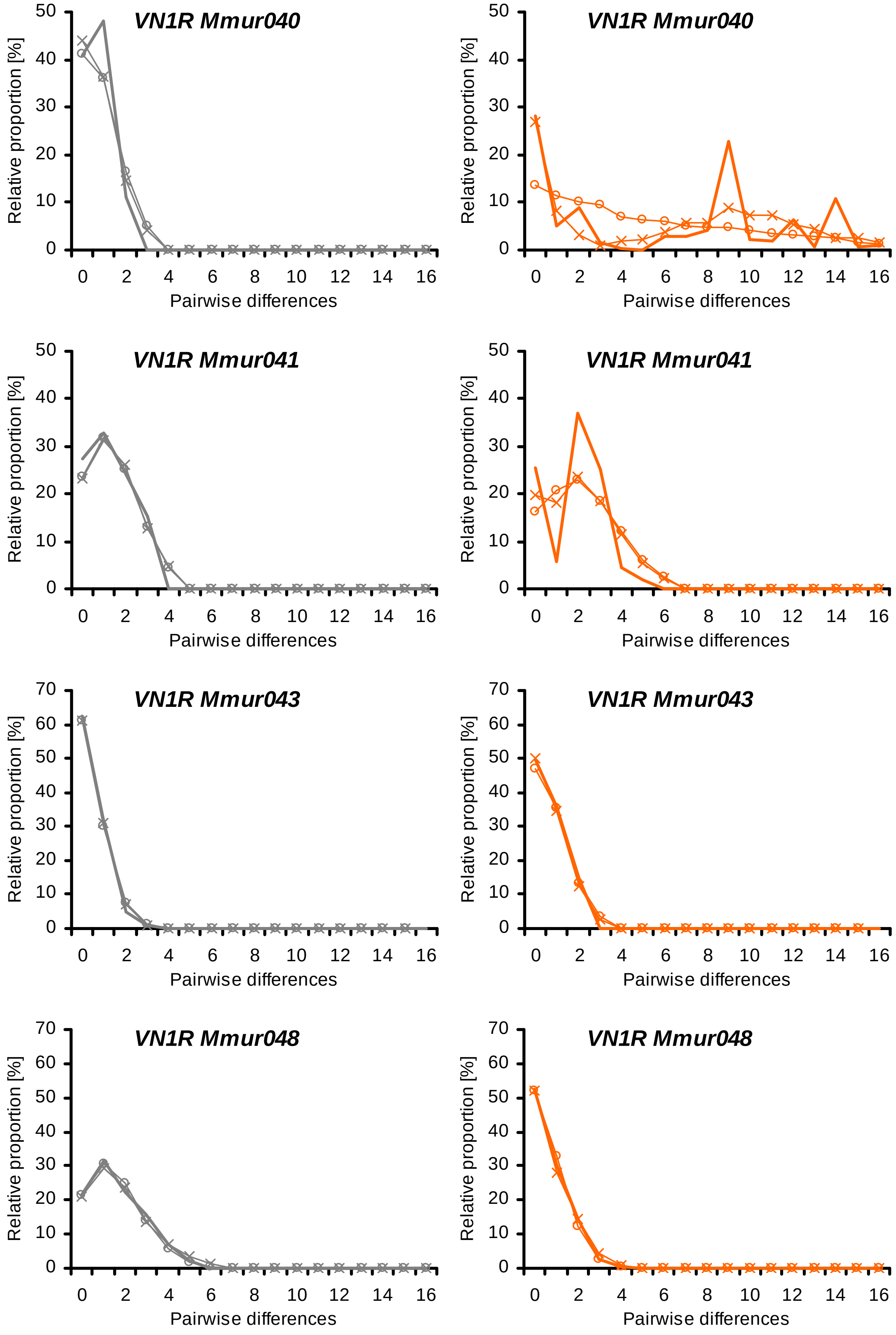

Additional file 2: *continued*

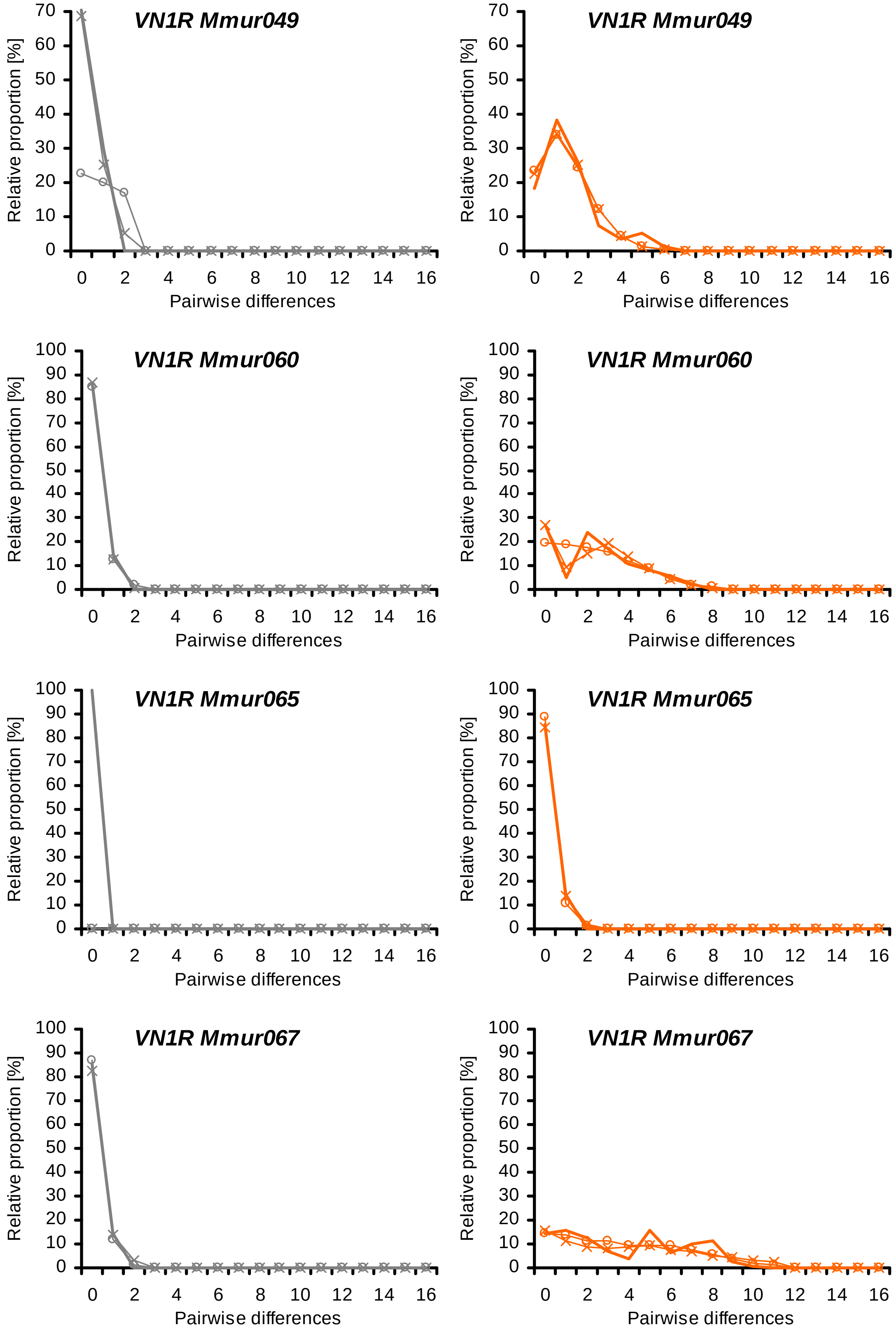

Additional file 2: *continued*

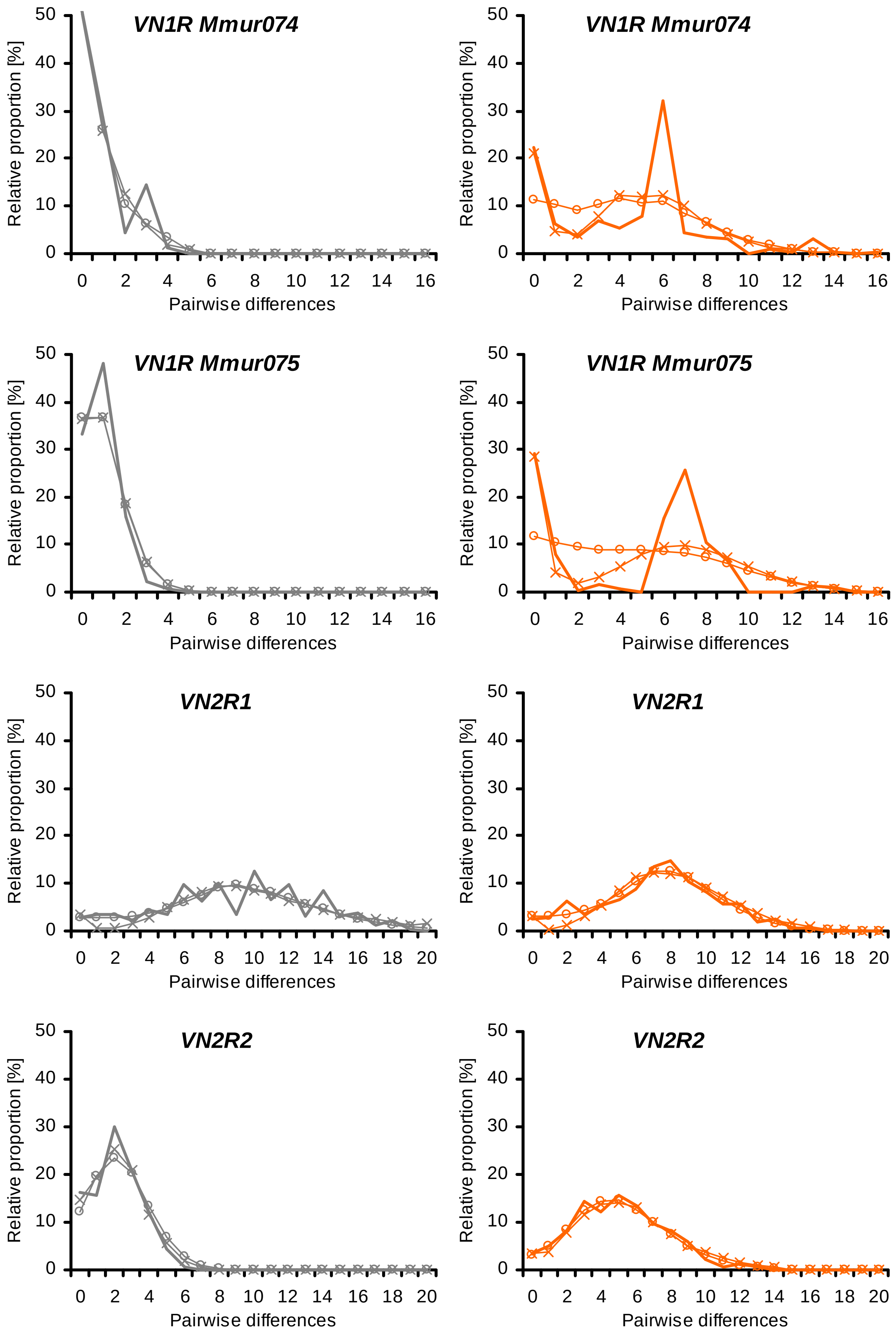

Additional file 2: *continued*
